# Health-related behavior as a mechanism behind the relationship between neighborhood social capital and individual health - a multilevel analysis

**DOI:** 10.1186/1471-2458-12-116

**Published:** 2012-02-10

**Authors:** Sigrid M Mohnen, Beate Völker, Henk Flap, Peter P Groenewegen

**Affiliations:** 1 Department of Sociology and Interuniversity Center of Social Science Theory and Methodology (ICS), Utrecht University, Heidelberglaan 2, 3584, CS Utrecht, The Netherlands; 2 NIVEL (Netherlands Institute for Health Services Research), Otterstraat 118 - 124, 3513, CR Utrecht, The Netherlands

## Abstract

**Background:**

Although various studies have found a positive association between neighborhood social capital and individual health, the mechanism explaining this direct effect is still unclear. Neighborhood social capital is the access to resources that are generated by relationships between people in a friendly, well-connected and tightly knit neighborhood community. We expect that the resources generated by cohesive neighborhoods support and influence health -improving behaviors in daily life. We identify five different health-related behaviors that are likely to be affected by neighborhood social capital and test these behaviors separately as mediators.

**Methods:**

The data set pertaining to individual health was taken from the 'health interview' in the 'Second Dutch national survey of general practice' (DNSGP-2, 2002). We combine these individual-level data with data from the 'Dutch housing demand survey' (WBO, 1998 and WoON, 2002) and statistical register information (1995-1999). Per neighborhood 29 WBO respondents, on average, had answered questions regarding social capital in their neighborhood. These variables have been aggregated to the neighborhood level by an ecometric methodology. In the main analysis, in which we tested the mediation, multilevel (ordered) logistic regressions were used to analyze 9253 adults (from the DNSGP-2 data set) from 672 Dutch neighborhoods. In the Netherlands, on average, neighborhoods (4-digit postcodes) comprise 4,000 inhabitants at highly variable population densities. Individual- and neighborhood-level controls have been taken into account in the analyses.

**Results:**

In neighborhoods with a high level of social capital, people are more physically active and more likely to be non-smokers. These behaviors have positive effects on their health. The direct effect of neighborhood social capital on health is significantly and strongly reduced by physical activity. This study does not support nutrition and sleep habits or moderate alcohol intake as possible explanations of the effects of neighborhoods on health.

**Conclusions:**

This study is one of the first to test a mechanism explaining much of the direct effect of small-area social capital on individual health. Neighborhood interventions might be most successful at improving health if they stimulate both social interaction and physical activity.

## Background

Contextual social capital and, in particular, small-area social capital (such as neighborhood social capital) affect health [1-5]. Neighborhood social capital can be defined as the access to resources that are generated by relationships between people in a friendly, well-connected and tightly knit community. Such communities are often referred to as 'cohesive communities'. Neighborhood social capital is the outcome of a cohesive community; in Coleman's formulation, it is the resource that "inheres in the structure of relations between actors" [6]. For example, enjoying a clean and safe playground that was organized and is supervised by the neighborhood is a resource produced by a cohesive neighborhood. One person alone would not have been able to achieve the same goal, even with a high level of human or financial capital. Neighborhood social capital is a public good and is available to all members of a community [6]. Neighborhoods differ in regard to this public good, which explains some aspects of differences in health between neighborhoods. Although scholars have found a positive association between neighborhood social capital and individual health, the mechanism explaining this direct effect is still unclear. Until now, it has been uncertain how neighborhood social capital affects an individual's health [7,8].

**Figure 1:**
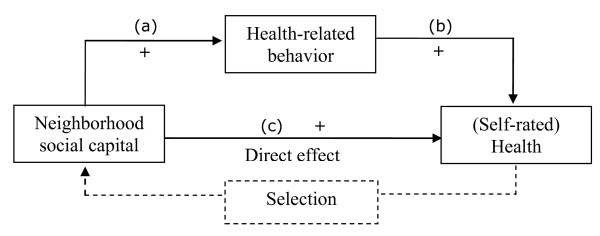
**Model of how neighborhood social capital may affect self-rated health**.

Activities undertaken to satisfy daily needs and leisure activities both start in the neighborhood; for example, the daily commute begins in the neighborhood. Daily needs can also be met entirely in the neighborhood, as by buying groceries in a neighborhood shop or by bringing children to a kindergarten in the neighborhood. At the end of a day, the neighborhood can be the site of recreational activities such as walks or gardening. These daily behaviors might explain how neighborhood social capital 'gets under the skin' [9] of inhabitants. Figure 1 illustrates a possible mediator effect in a traditional Baron and Kenny [10] path diagram. The direct effect presented in Figure 1 is as follows: the more neighborhood social capital, the better one's health (c). The mediator is the positive influence of neighborhood social capital on health-related individual behavior (a), which results in improved health, as in path (b).

Until now, few studies have focused on path (a). We present evidence from studies on subjects that diverge from our research interest because the data on this subject are rare. Cited studies thus differ in regard to the context, the operationalization of social capital, whether contextual social capital was measured at the neighborhood level or only at the individual level, and the health outcome variable.

Some studies indicate an association between contextual social capital and smoking [11,12]. A Swedish study found a negative association between individual-level social capital (operationalized as social participation in formal or informal groups in society) and daily smoking [11]. A multi-level study on 10,617 adults living in 19 urban and rural geographical areas (larger in size than our neighborhood units) in Minnesota, U.S.A., found evidence for a negative relationship between smoking and community social cohesion [12]. That study used measurements of social cohesion that were similar to our neighborhood social capital measurements.

Some studies link contextual social capital to alcohol consumption [13,14]. On the individual and contextual levels (27,687 students in 119 US colleges), Weitzman and Chen [13] showed that social capital (measured as voluntarism) was significantly negatively associated with several kinds of alcohol misuse. Another study showed that contextual social capital (voting behavior; 1.1 million people in 84 Finnish regions) decreases the risk of alcohol-related mortality [14].

Neighborhood social capital has been shown to stimulate physical activity in adults [15-17] and children [18-22]. A study from Melbourne, Australia (1,405 women in 45 suburban neighborhoods with an average of 4,000-30,000 inhabitants) showed that women who participated in local groups or events and (less consistently) women living in neighborhoods where residents trusted one another were more likely to participate in leisure-time physical activities [16]. A study on elderly people in Portland, U.S.A., also showed promising results (582 elderly in 56 neighborhoods). Neighborhood social cohesion, in conjunction with other neighborhood-level factors, was significantly associated with increased levels of neighborhood physical activity [17]. A study using data on the Dutch city Eindhoven and its surrounding areas (4,785 individuals in 213 small neighborhoods) showed no linear association between lack of participation in sports and neighborhood social cohesion [23] measured on the individual level. However, people living in medium social cohesion neighborhoods were more likely to participate in sports than inhabitants of low or high social cohesion neighborhoods. A study on 6,470 children in four Dutch cities showed that neighborhood social capital (measured at the individual level) was positively associated with outdoor play [18]. A study on 15 neighborhoods in Amsterdam found that inhabitants of neighborhoods where people do not know each other well tend to bicycle less often than people in other neighborhoods [15]. In summary, some research literature has already focused on the neighborhood as context and indicated that contextual social capital stimulates different kinds of health-related behavior.

To our knowledge, only three studies [4,24,25] have used behavior as the mediating factor (Figure 1, paths (a) and (b)) to explain the effect of contextual social capital on health (Figure 1, path (c)). First, Mohan et al. [24] showed that the direct effects of several different small-area measurements of social capital on mortality became weaker once health-related behaviors were included in the models. As the authors note, however, mortality might be an insufficiently sensitive indicator of individual health. Subjective health (self-rated health) is a broader measure. Self-rated health is well established as an indicator of morbidity [26] and a predictor of mortality [27], and it is more responsive to recent events than other measures. Furthermore, to understand the mediating effect of health-related behavior, behaviors should not be considered all together, as in the study by Mohan et al. Neighborhood social capital might influence different health behaviors in different ways. For example, large quantities of alcohol are often consumed in groups; a well-connected neighborhood might give more opportunities for group drinking than un-connected neighborhoods. At the same time, a well-connected community might disapprove of smoking. The second study that tested behaviors as mediators also considered all behavior mediators together [4]. Poortinga studied the association between neighborhood social capital and self-rated health using a British data set and found no mediation effect. The third study [25] analyzed behaviors separately. While changes in exercise, smoking or weight loss were positively associated with individual-level community belonging, changes in alcohol consumption and taking vitamins were not. Some limitations of this study are the measure of community belonging solely on the individual level and the focus on changes in behavior, rather than behavior itself. Moreover, large regions (up to 2.5 million people per region) were used.

In conclusion, it is not clear whether different kinds of health-related behaviors are mediators of the association between contextual-level social capital and individual health. Physical activity seems to be a promising mediator because evidence on the effects of contextual social capital on physical activity is, in comparison to studies on other mediators, the best developed; however, it has not yet been studied as a mediator in a neighborhood study on health. Our study answers the research question: *Do health-related behaviors explain the association between neighborhood social capital and individual health?*

### Neighborhood social capital and individual behavior

Neighbors live close to each other, and therefore, it is likely that neighbors observe and learn from each other's behavior [28,29], especially if the individuals involved are strongly socially connected. It can be argued that personal contacts might be easier in the countryside, where every individual knows everyone else from childhood and by name. Behavior that does not conform to the norms of the community might be sanctioned more efficiently in the countryside than in cities because rural inhabitants have fewer alternative opportunities for social contacts. Urban people, however, can also be affected by social capital. Neighbors in cities have more opportunities for daily contacts because they live very close to each other. People who live close might provide 'feedback', which is essential for developing social behaviors [30]. Norms of behavior are provided by a community and not given by one or two close friends only [9]. Behavior is a result of internalized community norms, imitation, and social feedback.

If neighborhoods differ in regard to their level of social capital, the effects of norms on inhabitant's behavior will differ between neighborhoods as well. As argued above, focusing on specific behaviors is a necessary strategy to identify how contextual factors may improve health. This approach is especially valuable for prevention strategies and promotion of healthy lifestyles. This study distinguishes five health-related behaviors associated with a healthy lifestyle [31,32]. Individual health is related to smoking, drinking, sleeping, and eating habits as well as to physical activity. Neighborhood communities might differentially affect these behaviors because a given behavior may be more common in some neighborhoods than in others. Moreover, some behaviors might be easier to disapprove of than other behaviors. We assume that health-related behavior is beneficially affected by neighborhood social capital. For example, a well-connected community with a common sense of health-related norms might disapprove of smoking. Second, a community with a high level of social capital might intervene or report underage drinking to the parents [33]. Third, people's sleeping rhythms may adjust to coincide with the time when the lights are switched off in their neighbors' houses. Fourth, patterns of food consumption might also be influenced by the neighborhood (e.g., through the smell of dinners being prepared). Fifth, physical activity might be affected by neighborhood norms as well [17]. Physical activity refers not only to sports (e.g., soccer or jogging) but also to walking and biking for relaxation or transportation.

We are aware that the positive influence of a well-connected community on behavior is only an assumption. We exclude the possibility that behavior might also be negatively affected by neighborhood norms. For instance, a cohesive neighborhood might provide more opportunities for alcohol consumption, and if community norms trivialized risky behaviors such as driving under the influence of alcohol, the risk of an alcohol-related accident or alcohol addiction would be increased. Norms are difficult to measure and were not included in the data used in this article. In an attempt to compensate for this gap in our knowledge, we tested in pre-analyses whether the religiosity - as an indicator for norms of moderateness- of a neighborhood is an indicator for healthy behavior. We did not find a religiosity effect on health-related behavior, and no interaction of religiosity with neighborhood social capital as influences on health was found. Therefore, we present our analysis without an indicator for health-related norms. To analyze the mediation effect of behavior, we test for each health-related behavior separately whether *more neighborhood social capital is associated with more of that health-related behavior*.

### Behavior resulting in health

The extent to which neighborhood social capital affects health via behavior depends on the degree of influence behavior has on health (Figure 1, path (b)). Fortunately, a wealth of research confirms that certain behaviors affect health. Tobacco consumption, for example, is associated with morbidity and mortality. A British longitudinal study on physicians showed that non-smokers had a 10-year longer life expectancy than smokers [34]. Moderate alcohol consumption is positively associated with subjective health in contrast to no or excessive alcohol consumption [35]. A review by Alvarez and Ayas [36] showed that a daily sleep routine of 7 to 8 h promotes health, as measured by all-cause mortality. Irregular breakfasts have been shown to be an important risk factor for overweight and obesity in adolescents [37]. One warm meal per day is also advised [38]. Regular physical activity is associated with lower morbidity and mortality rates [39]. In summary, the literature shows that *non-smoking, moderate alcohol consumption, seven or eight hours of sleep per night, regular breakfasts, warm meals, and physical activity are related to good health*.

The direct effect shown in Figure 1 might be explained by behavior and its effect on individual health. The mediation might emerge fully or only partly because, along with behavior, other mechanisms (e.g., psycho-biological explanations or access to facilities) are also responsible for shaping health. To answer our research question, we analyze whether *the effect of neighborhood social capital on health is (partly) mediated by health behaviors*.

In this article, the moderation hypothesis illustrated in Figure 1 was tested step-by-step. First, the effect of neighborhood social capital on five different health-related behaviors was tested. If a relationship was found, the strength of the behavior's association with self-rated health was reported. Finally, each of these behaviors was tested for whether it weakened the associations between neighborhood social capital and health.

## Methods

### Data set

This study used three different survey data sets, as well as register information. The individual data set was from the 'health interview' in the 'Second Dutch national survey of general practice' (DNSGP-2, 2001/2002) [40], which was designed by the Netherlands Institute for Health Services Research (NIVEL) and the Dutch National Institute for Public Health and the Environment (RIVM). We combined these individual-level data with two data sets from the 'Dutch housing demand survey' (WBO, 1998 and WoON, 2002), which were collected under the supervision of the former Ministry of Housing, Spatial Planning, and Environment (VROM), and statistical register information collected by Statistics Netherlands (1995-1999). The datasets were merged based on the respondent's 4-digit postal code (neighborhoods). In the Netherlands, 4-digit postal codes are relatively small units; they comprise areas between 1 and 8 km^2^, with on average 2,500-3,000 addresses and about 4,000 residents. In our study, we used 672 postcodes ranging from 140 to 31,620 inhabitants (with on average 6,908 residents).

The 'health interview' is a 5% random sample of listed patients in 104 Dutch general practices (12,699 individuals; response rate of 65%). In the Netherlands, nearly all people are on the list of a specific general practitioner or practice, irrespective of their health status. Practices were sampled according to region, urban status, and practice type. The "health interview" in the DNSGP-2 contains several health and health-related measurements. Interviews were conducted at the homes of the respondents.

The WBO 1998 data set evaluates physical and social aspects of housing in the Netherlands. WBO 1998 is representative of all Dutch people 18 years or older. Sample selection was conducted using municipal registration information, and the data thus cover 117,569 people (response rate = 78%). Questions regarding neighborhood social capital were only asked of the heads of the household because it was expected that only they would be able to answer housing-specific questions.

To include the information on whether social capital has changed over time, the WoON 2002 data set, also a cross-sectional survey and the successor of the WBO 1998, was used. It incorporates the same neighborhood social capital variables as the WBO 1998. WoON 2002 includes data on 75,043 individuals (response rate = 61%).

Statistics Netherlands offers free register information on socio-demographic information regarding Dutch 4-digit postal code areas. Under Dutch privacy legislation, for survey research among the general population, no further research ethics approval is required.

Of the 12,699 respondents of the DNSGP-2 data set, only 9684 were adults. Moreover, we lost cases because of missing values in individual control variables (n = 33) or in behavior variables (n = 42). The neighborhood-level analysis caused further case loss (neighborhood social capital, n = 184; neighborhood income, n = 172). In this study, 9,253 respondents from the DNSGP-2, living in 672 neighborhoods, were used. Analyses have shown (in data not presented here) that the cases lost did not change the representative quality of the adult data sample.

### Measurements

#### Individual variables

All individual variables were (Table 1) generated from the DNSGP-2 'health interview'.

**Table 1:** A descriptive table of individual and collective variables

n_i _= 9253., n_j _= 672		*Range*	*Mean*	*S.D*.	*Valid Percent*
*Individual level*					
					
Self-rated health:	good or better				81.2%
	not good				18.8%
Gender:	woman				55.5%
Age in years		18 - 97	48.9	17.1	
Nationality:	Dutch				98.1%
	Non Dutch				1.9%
Education	low				48.8%
	middle				26.5%
	high				24.7%
Having a paid job	Student				4.2%
	Housewives /-men				20.8%
	(Self-) employed				50.4%
	Registered unemployed				1.4%
	Incapable of working				5.5%
	(Invalidity) pensioner				17.7%
Household equivalent income per person /100	Missing category low				6.7%
					32.1%
	middle				33.8%
	high				27.4%
*Health-related behavior*					31.2%
	Smoker				
Smoking status					
	Ex-/Never smoker				68.8%
Alcohol intake	*(almost) never (0-3 glasses) *				57.4%
(glasses per week)	Moderate (4-11)				21.4%
	*High (> 11)*				21.2%
Sleep duration	≤ 6 hours				19.8%
	7-8 hours				69.5%
	≥ 9 hours				10.8%
Nutrition	1 warm meal/day & ≥ 5x breakfast per day				76.0%
	Less often				24.0%
Physical activity	≥ 5 times per week 30 min.				57.7%
	less often				42.3%

*Neighborhood level*					
Neighborhood social capital		-0.77 - 0.54	-0.08	0.214	
Neighborhood social capital change 2002-1998		-0.61 - 0.56	0.08	0.175	
Percentage of rich residents (in %)		5 - 54	17.61	7.205	
Home maintenance		2-5	3.95	0.388	
Urbanity of the municipality		1-5	3.35	1.288	

Note: n_j _= neighborhood; n_i _= individuals; 13.8 people per neighborhood (range 1-277).

*The main outcome variable *was 'self-rated health', with the following possible answers: 'excellent/very good/good/fair/bad'. The original, skewed scale was dichotomized, with (1) representing excellent to good health and (0) representing fair or bad health.

The *Individual control variables *were sex, age, nationality, and social status. *Women *were indicated by (1) and men by (0). *Age *was measured in years and centered on the mean (48.9). *Nationality *was a dummy variable, with Dutch (1) and Non-Dutch (0) nationality as answer categories. Social status was measured by education, employment, and income. *Education *was measured by the highest level of education attained, in three categories: low (1), middle (2), and high (3). *Employment *was measured with six possible answer categories: 'student', 'housewives/-men and others', 'registered unemployed', '(self-) employed', 'incapable of working', and '(invalidity) pensioner'. *Income *was presented as the household equivalent income per person, collapsed into three categories from low (1) to high (3), with a category for 'missing values'.

##### Health-related behavior

Five measurements of health-related behavior were considered in this study. First, the three-category variable *smoking status *was collapsed into non- and ex-smoker (1) versus current smoker (0). Second, '*alcohol intake*' was measured by asking separately the alcohol intake in number of glasses during the week and during the weekend. The answers were summed up to a week-score of alcohol intake in glasses per week. The relationship between alcohol consumption and self-rated health is curve-linear; the more alcohol an individual consumed, the better his or her health, until the trend reverses and the relationship becomes negative. No alcohol intake (0 g/week alcohol) is suboptimal for health; moderate alcohol intake (> 0 and < 200 g/week alcohol) is optimal, and a high alcohol intake is negatively associated with health (> 200 g/week alcohol) [41]. We studied this particular relationship using our own data. As a result, we collapsed the number of glasses of alcohol consumed in the last week into no or almost no alcohol intake (0-3 glasses per week), moderate alcohol intake (4-11 glasses per week), and high alcohol intake (12 or more glasses per week). Third, *sleep duration *was measured by the survey question "How many hours do you sleep?". We tested the association between sleep and self-rated health and found (in accordance with existing literature [36]) that fewer or more than 7 or 8 h is negatively associated with health. Sleep duration was collapsed into a healthy sleep duration of 7 to 8 h (= 1), and two unhealthy sleep durations of less (= 0) or more than 7 to 8 h (= 2). Next, *nutrition habits *were measured by asking two questions: the respondent was asked how many days per week he or she had breakfast and whether the respondent had at least one warm meal per day. Nutrition habits were considered healthiest if breakfast was eaten '5 to 7 times per week' and if the respondent consumed one warm meal per day. Most Dutch people have one warm meal a day. Nutrition habits were added as a dummy variable, where (1) indicates the consumption of one warm meal per day and breakfast more than four times per week; otherwise, nutrition habits are coded as (0).

Finally, *physical activity *was measured by asking: *"*On how many days do you do 'activity X' for at least thirty minutes?". Physical activities included biking, doing odd jobs, gardening, sports, or other physical activities [38]. In general, physical activity is positively associated with self-rated health. For adults, it is advised that they be physical active for at least 30 minutes, 5 days a week [39]. Additional analysis of our own data had confirmed this health advice. Thus, five or more days of 30 minutes of activity was coded with (1); less physical activity was coded with (0).

#### Neighborhood variables

*The core independent variable *is the neighborhood social capital, as determined from the WBO 1998 data set. An average of 29 respondents per neighborhood was used to estimate the neighborhood social capital. Neighborhood social capital is measured by three questions pertaining to contact among neighbors. The questions ask 1) whether people in the neighborhood know each other, 2) whether neighbors are nice to each other, and 3) whether there is a friendly and sociable atmosphere in the neighborhood. Response categories were 'totally agree', 'agree', 'neutral', 'don't agree', and 'totally don't agree' on a scale of 1 to 5. To aggregate the individual information to the neighborhood level, we use ecometric measurements [42-44], as in earlier work [2], by performing a multilevel analysis. This approach accounts for the nesting of social capital items within individuals and includes the neighborhood level in the analysis, resulting in a three-level model (neighborhoods, individuals, and the items measuring social capital). We adjusted for seven individual characteristics that may influence the perception of neighborhood social capital: sex, age, education, income, employment status, home ownership, and years of residence. The ecometric model also accounts for differences in the numbers of respondents per neighborhood by shrinking deviating neighborhoods with smaller numbers of respondents to the general average [45]. The residuals of the neighborhood social capital measurement, i.e., the part that cannot be attributed to individual response patterns and measurement error, constitutes the social capital measurement. Positive values indicate higher than average levels of neighborhood social capital (reliability based on Hox [45]:0.707).

We argued above that the outcome variable of the main analysis (self-rated health) is very sensitive to recent developments. Neighborhood social capital might have changed between the time it was last measured, in 1998, and when self-rated health was measured in 2002. To control for the possibility of an increase or decrease in the level of social capital in a neighborhood over these 4 years we calculated a change score. Fortunately, the same measures of neighborhood social capital used for 1998 are available for 2002. We first calculated neighborhood social capital for 2002 in precisely the same way as for 1998 (reliability based on Hox [45]: 0.720). Subsequently, we computed a change score by subtracting neighborhood social capital in 1998 from neighborhood social capital in 2002. A positive value of the change score indicates an improvement, and a negative change score indicates a decline in neighborhood social capital. Two-thirds of the neighborhoods had not changed, staying within one standard deviation from the mean (0.08) of the change score.

Three control variables at the neighborhood level were used (Table 1). To take into account the level of income in a neighborhood, we used the percentage of people in the *highest income quintile*. Hou and Myles [46] showed that the prosperity of a neighborhood is associated with the inhabitants' health and that this effect is even stronger than the effect of poverty. The data from 1997 were provided by Statistics Netherlands. If information was missing, we used data from 1995 or 1999 instead.

Next, we used the degree of *urban density of the municipality *in which a given neighborhood was located in 1999. The codes were provided by Statistics Netherlands and were based on the number of addresses per km^2 ^(1 = rural = up to 499 addresses per km^2^; 2 = semi-rural = 500-999 addresses/km^2^; 3 = intermediate urban/rural = 1,000-1,499 addresses/km^2^; 4 = semi-urban = 1,500-2,499 addresses/km^2^; and 5 = urban = more than 2,499 addresses/km^2^).

Finally, we used a measure of *home maintenance in the neighborhood *to control for aesthetic/physical environmental influences on health. The variable is aggregated (via the mean) to the neighborhood level. On average, 29 people per neighborhood answered this question. Maintenance was addressed in the WBO 1998 with the question, 'Is your house in a bad condition?'. Answers were on a scale from 'I totally agree' (1) to 'I totally do not agree' (5). Higher values thus indicate better maintenance, as reported by the respondent.

### Analytic strategy

To test the association and mechanism of association between neighborhood social capital and self-rated health, we performed multi-level logistic regression analyses. We estimated the models with the statistical software package Stata 11, using the command *xtmelogit*. Our study design is in the tradition of Baron and Kenny [10], and it meets the requirements of a multi-level mediational model [47]. The first multi-level logistic regression analyses were used to determine whether social capital has an effect on healthy behavior (Table 2). Two of these analyses had to be ordered because the dependent variables had three instead of two categories. We report only the coefficient of interest, the category we expect to be health-improving. Next, analyses were conducted to determine whether these behaviors improve health (Table 3, 1-2). When we had found that these behaviors were related to health and that they were also significantly positively associated with neighborhood social capital, we conducted multi-level logistic regression analyses to determine whether these mechanisms mediate the relationship between the neighborhood social capital and health (Table 3 Model 4-5). The direct link between the neighborhood social capital and health is also presented in Table 3 (Model 3). This neighborhood social capital effect on self-rated health, presented in Model 3, can be compared with the neighborhood social capital variable presented in Models 4 and 5. A decrease in the coefficient of the neighborhood social capital variable indicates a mediator effect of the tested health-related behavior. We tested the significance of the mediation with the product-of-coefficients approach [48], also called the Sobel test [49]. We used Preacher's Sobel test webpage [50], which also meets the requirements to test a multi-level mediation effect [47]. We were aware of the possible problems in the estimation, when doing mediation model linearly when the paths are non-linear. However, given the strength of the effects found and our large sample size, the risk of inadequacy is small.

## Results

Table 1 shows that more than 80% of the Dutch reported good or very good self-rated health. The sample shows an overrepresentation of housewives/-men in comparison to the percentage in the Netherlands as a whole (8% in 2006). Regarding the characteristics of the study population and the neighborhoods, Table 1 shows that two-thirds of those surveyed reported themselves to be non-current smokers. More than 20% of the respondents drank alcohol in moderation. Two-thirds of the participants slept 7 or 8 h per night, and even more reported healthy eating habits. Furthermore, 58% of the participants reported being physically active five to seven days per week for at least 30 min per day.

### Neighborhood social capital and health-related behavior

Table 2 shows the results of analyses for different health-related behaviors; each model has a different behavior as the dependent variable. Table 2 Model 1 shows that the association between neighborhood social capital and being a non-smoker is positive; this association is significant. Model 2 shows that the likelihood of moderate alcohol intake is slightly but not significantly reduced by high neighborhood social capital. Model 2 and Model 3 were based on an ordered regression. Models 3 and 4 in Table 2 suggest that healthy sleep patterns and eating habits are not affected by the neighborhood's level of social capital. The strongest association between the neighborhood social capital and a health-related behavior is presented in Model 5, Table 2. People living in neighborhoods with a high level of social capital have a 118% greater chance of being physically active than people living in low social capital neighborhoods.

**Table 2:** Multilevel logistic regression analyses of neighborhood social capital on five health-related behaviors (Odds Ratios, 95% Confidence Interval in parentheses)

n_i _= 9253, n_j _= 672	Model 1	Model 2 (ordered)	Model 3 (ordered)	Model 4	Model 5
***Dependent variables***	**Non-smoker^a^**	**Moderate alcohol intake^b^**	**7 or 8 h sleep duration^b^**	**Healthy nutrition pattern^c^**	**≥ 5 times per week 30 min. physical activity^d ^**

Neighborhood social capital	1.54 (1.08/2.19)	0.96 (0.93/1.00)	1.02 (1.00/1.05)	1.27 (0.86/1.86)	2.18 (1.26/3.80)

Neighborhood variance (estimate)	0.026 (0.014)	0.028 (0.019)	0.00 (0.000)	0.036 (0.020)	0.342 (0.049)
ICC, %	0.8	0.01	0.0	1.1	9.4

Note: n_j _= neighborhood; n_i _= individuals. All models are controlled for individual (age, sex, nationality, and SES) and neighborhood (highest income quintile, urbanity of the municipality, home maintenance in the neighborhood) level characteristics as well as change of neighborhood social capital between 1998 and 2002. ^a^Reference category: ex- or current smoker. ^b^An ordered logistic regression multi-level analysis was performed. ^c^Reference category: unhealthy pattern. ^d^Reference category: less often.

The mechanism (a) from Figure 1 applies to two behaviors: the more neighborhood social capital, the more physically active the residents are and the more likely it is that they are non-smokers. The other three behaviors cannot serve as mediators because of the non-significant association with neighborhood social capital. We continued our analysis with only these two behaviors.

### Non-smoking, physical activity and better health

Table 3 Model 1 shows that non-smoking status is positively associated with self-rated health. Physical activity is similarly, but more strongly, associated with self-rated health (Table 3 Model 2). The likelihood of 'good' or 'very good' health is almost doubled by physical activity.

**Table 3:** Multilevel logistic regression analyses of the mediator effect of health-related behavior, dependent variable self-rated health (Odds Ratios, 95% Confidence Interval in parentheses)

n_i _= 9253, n_j _= 672	Model 1	Model 2	Model 3	Model 4	Model 5
Neighborhood social capital			1.75 (1.10/2.78)	1.71 (1.08/2.72)	1.58 (1.01/2.47)
Non-smoking	1.25 (1.10/1.42)			1.24 (1.09/1.41)	
Physical activity		1.95 (1.73/2.19)			1.94 (1.73/2.18)

Variance neighborhood level (estimates and s.e.)	0.068 (0.030)	0.047 (0.028)	0.057 (0.029)	0.056 (0.029)	0.038 (0.026)
Intra class correlation (%)	2.0	1.4	1.7	1.7	1.1

Note: n_j _= neighborhood; n_i _= individuals. Variance of the empty model was 0.161 (0.036). All models are controlled for individual (age, sex, nationality, and SES) and neighborhood (highest income quintile, urbanity of the municipality, home maintenance in the neighborhood) level characteristics. Model 3 to 5 are additionally controlled for change of neighborhood social capital between 1998 and 2002.

### The mediating effect of health-related behavior

**Figure 2:**
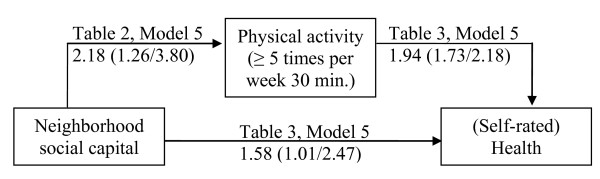
**Physical activity as a mediator of the association between neighborhood social capital and self-rated health**.

Table 3 Model 3 shows that neighborhood social capital is positively associated with health. This finding is the direct effect of neighborhood social capital on self-rated health. Table 3 Model 4 shows that this direct effect is only slightly reduced by the variable 'non-smoking'; the Sobel test shows that non-smoking status is not a significant mediator (*p *= 0.740). The most interesting finding from Table 3 is presented in Model 5: the direct effect presented in Model 3 is considerably attenuated by physical activity; the Sobel test shows that physical activity is a significant mediator (*p *= 0.007). Figure 2 summarizes the findings (Odds ratios, 95% Confidence Interval in parentheses) on physical activity as mediating the influence of social capital on health: the direct effect of social capital on health becomes weaker if physical activity is included in the model.

## Discussion

In neighborhoods with a high level of social capital, people are more physically active and more likely to be non-smokers. These behaviors have a positive effect on self-rated health. Moderate alcohol intake, nutrition, and sleep habits did not explain why neighborhood social capital is associated with self-rated health.

Physical activity is the behavior that is most sensitive to influence by characteristics of the neighborhood. This association might be strongest because physical activity usually occurs in a public space, while eating breakfast and dinner as well as sleeping are private, indoor activities. Drinking and smoking happen both indoors and outdoors, making them both visible and invisible to neighbors. Therefore, these behaviors are not particularly strongly linked to the neighborhood context. Furthermore, the ease of interpreting behavior as healthy may depend on the kind of behavior. While it is common knowledge that non-smoking and regular physical activity are healthy, it might not be clear how many hours of sleep promote health. Healthy behaviors that are less clearly defined are more difficult to promote or evaluate.

The results of our study are consistent with the previously mentioned British study (7,394 adults, 720 neighborhoods) with regard to only one effect: neighborhood social capital has a positive effect on non-smoking status [4]. None of the behavioral variables in Poortinga's study [4] functioned as a mediating variable and the direct association between community social capital and health even increased instead of decreased. Poortinga had lumped together all available behaviors as mediators, including those with no significant association with social capital. A separate test of each behavior might have shown different results. Furthermore, only significant and positive associations (Figure 1, mechanism (a)) should be used as mediators. In an additional analysis (not shown), we found that adding alcohol consumption (which was negatively and non-significantly associated with neighborhood social capital) to the model as a single behavioral mediation variable increased the association between neighborhood social capital and health.

Our findings are consistent with the findings of Mohan et al. [24], who found that the direct effect of small-area social capital on mortality was attenuated by health-related behaviors [24]. We built on these findings by using self-rated health as a dependent variable, observed values of social capital instead of estimates, separate analyses for each behavior instead of analyzing all behavior mediators at once, and finally, data from the Netherlands (instead of Great Britain, as in [24] and [4]).

In contrast to the existing literature [13,14] and to our predictions, our study does not confirm that the contextual social capital is significantly and positively associated with moderate use of alcohol.

An unexpected finding is that short-term changes in neighborhood social capital is also positively associated with self-rated health (Additional file 1, Model 1, odds ratio (confidence interval): 1.69 (1.10/2.61)). We used change in neighborhood social capital as a control variable because our measure of neighborhood social capital came from a data set collected 4 years before the (comparable) data set that measured the outcome variable and the individual level controls. This finding indicates that changes in neighborhood social capital are as important for health as the current level of social capital. Future research should pay attention to the effects of these dynamics in social capital.

Our study has a limitation in regard to a possible third neighborhood factor that might influence both neighborhood social capital and physical activity. For instance, a neighborhood can be built in such a way that it promotes both physical activity *and *social interaction [51]. Lund [52] studied the effect of the built environment on health by comparing two different neighborhood types in the U.S. city of Portland, Oregon. The more walk-friendly neighborhood showed a greater sense of community; However, Lund's study was limited because only two neighborhoods were compared. Cohen et al. [51] had found that "parks within various distance to one's tract" (page 201-2; tract = neighborhood) were positively associated with collective efficacy on the individual level. The authors had interpreted the collective efficacy as an indicator of neighborhood social capital. The study analyzed data on 65 neighborhoods in Los Angeles, California. Cohen's work indicates that parks have the potential to increase inhabitants' health in multiple ways: parks in the neighborhood provide incentives for physical activity and social interaction, and the green-space itself might increase well-being and lower stress [53]. Recent studies on *neighborhood walkability *have shown that the built environment affects physical activity; it can also be assumed that a 'walk-able' neighborhood that stimulates, for example, walking for recreation [54], might also affect the social environment.

Independent of whether a third environmental factor might be involved, it cannot be ruled out that social capital be increased *by *physical activity. Neighbors who go for a walk with the dog or play with their children at the playground have more meeting opportunities than inactive inhabitants.

Most research conducted in this area cannot rule out reversed causality between the dependent variable and the main explanatory variable. It might be that bad health would hinder interaction with neighbors; systematically, this effect could result in fewer chances to build neighborhood social capital. For this study, longitudinal individual data were not available to test reversed causality; however, neighborhood social capital, the influencing variable, was measured before the dependent health variable. A further limitation is that it might be possible (and impossible to account for in this study) that people who like physical activity (who, for example, do their grocery shopping by bicycle) chose neighborhoods with particular physical characteristics [55]. Future research will determine to what extent the sense of community in the neighborhood, the built environment, the clustering of physically active inhabitants, or neighborhood selection affects health via the mediation factor "physical activity".

Our study is an important contribution to research on mechanisms explaining associations between effects at the micro and macro level. Until now, few studies inquire into multi-level mediation effects. Moreover, our study advances the empirical literature on social capital and health. One advantage of using more than one data source is that this study does not suffer from a 'single -source bias'. The data source used to measure health was not the same as the source used for social capital. Therefore, a third individual factor (e.g., a psychological link) cannot be the underlying cause for the association between social capital and health. Our study differs from previous examinations of this subject because the curve-linear associations between health and sleeping, as well as health and drinking behavior, were studied; categories were chosen carefully, and ordered logistic multi-level regression analyses were done when needed. By means of these approaches, our study improves upon existing literature [4], which used dichotomized mediating variables; such variables might have been too crude as measurements because of the inherent loss of reliable information and consequent difficulties with interpretation of the data.

This study showed that the relationship between neighborhoods and health is only partly explained by physical activity. Aside from physical activity, other mechanisms are also discussed in the literature. For instance, a well-connected neighborhood might lobby more effectively for a walk-friendly [56] and green neighborhood [57] or access to health care facilities [58] and healthy food [59]. The feelings of 'belonging to a (friendly) community' might benefit health via a psycho-biological pathway. For example, such feelings might lower blood pressure, decreasing the chance of coronary artery diseases and susceptibility to infectious diseases [9].

Ultimately, we would like to frame our findings on physical activity as a mediator in terms of health environment research. For years, research on physical activity had been limited to individual characteristics [60]. Recently, more attention has been given to the broader context (the physical and, even more recently, the social environment) in which the physical activity of individuals occurs [61]. Our study bolsters the importance of the social component of this ecological perspective [62], i.e., social capital.

## Conclusions

It seems that cohesive neighborhoods share health-related norms that are related to physical activity and that this characteristic explains much of the direct effect of neighborhood social capital on health; however, we cannot exclude the possibility that neighborhoods generate a high level of social capital *because *the residents are active and thus are more frequently in public spaces. Such an effect would suggest that it is not social capital but the collective physical activity that causes health differences between neighborhoods. Other mechanisms such as 'well-being', feeling attached to a neighborhood, and better access to facilities would not then play a role in differences in health between neighborhoods. These mechanisms were not tested in this study; however, these alternative mechanisms cannot be excluded because the direct effect was not completely explained. Future research should study not only alternative mechanisms but also alternative health outcome variables. The mediator 'physical activity' might be less significant in attenuating the direct effect between neighborhood social capital and, for example, mental health, than self-rated health.

Interventions aiming to increase both social interaction and physical activity are likely to be successful at improving health. Interventions should be accompanied by evaluations to disentangle the directions of causality.

## Competing interests

The authors declare that they have no competing interests.

## Authors' contributions

All authors have made significant contributions to this paper. SMM was the main author of the manuscript and was involved in all aspects of this paper. BV, HF, and PPG contributed equally to the conception, design and to the interpretation of this study. All authors read and approved the final manuscript.

## Pre-publication history

The pre-publication history for this paper can be accessed here:

http://www.biomedcentral.com/1471-2458/12/116/prepub

## Supplementary Material

Additional file 1 **Model 5 of Table 3 *with *control variables**. Click here for file
