# Supplementary material for: Health-related behavior as a mechanism behind the relationship between neighborhood social capital and individual health - a multilevel analysis
Source: BMC Public Health. 2012 Feb 10;12:116. doi: 10.1186/1471-2458-12-116 (PMC3347984; doi:10.1186/1471-2458-12-116)
Supplement: Additional file 1 — Model 5 of Table 3 with control variables. [file 1471-2458-12-116-S1.DOC]

Additional file:

| Odds Ratios, 95% Confidence Interval in parentheses. | | | |
| --- | --- | --- | --- |
| n_i_ = 9253,  n_j_ = 672 |  |  | Model 1 |
| Gender: | woman |  | 0.76 (0.66/0.86) |
| Age in years (centered) |  |  | 0.99 (0.98/0.99) |
| Nationality: | Dutch |  | 2.38 (1.64/3.43) |
|  | Non Dutch |  | (ref.) |
| Education | low |  | (ref.) |
|  | middle |  | 1.49 (1.28/1.74) |
|  | high |  | 1.76 (1.48/2.08) |
| Having a paid job | Student |  | 1.08 (0.72/1.64) |
|  | Housewives /-men |  | 0.71 (0.59/0.86) |
|  | (Self-) employed |  | (ref.) |
|  | Registered unemployed |  | 0.36 (0.24/0.54) |
|  | Incapable of working |  | 0.09 (0.07/0.11) |
|  | (Invalidity) pensioner |  | 0.58 (0.46/0.72) |
| Household equivalent income per person / 100 | Missing category |  | 0.84 (0.66/1.08) |
|  | low |  | 0.72 (0.62/0.83) |
|  | middle |  | (ref.) |
|  | high |  | 0.99 (0.84/1.17) |
| Neighborhood-level variables |  |  |  |
| Percentage of rich residents (in %) |  |  | 1.01 (1.00/1.02) |
| Home maintenance |  |  | 1.17 (0.93/1.46) |
| Urbanity of the municipality |  |  | 1.00 (0.93/1.07) |
| Neighborhood social capital change 2002-1998 |  |  | 1.69 (1.10/2.61) |
| Neighborhood social capital |  |  | 1.58 (1.01/2.47) |
| Physical activity |  |  | 1.94 (1.73/2.18) |
| Variance neighborhood level (estimates and s.e.) |  |  | 0.038 (0.026) |
| Intra class correlation (%) |  |  | 1.1 |

Model 5 of Table 3 with control variables:
